# Vaccination to Conserved Influenza Antigens in Mice Using a Novel Simian Adenovirus Vector, PanAd3, Derived from the Bonobo *Pan paniscus*

**DOI:** 10.1371/journal.pone.0055435

**Published:** 2013-03-11

**Authors:** Alessandra Vitelli, Mary R. Quirion, Chia-Yun Lo, Julia A. Misplon, Agnieszka K. Grabowska, Angiolo Pierantoni, Virginia Ammendola, Graeme E. Price, Mark R. Soboleski, Riccardo Cortese, Stefano Colloca, Alfredo Nicosia, Suzanne L. Epstein

**Affiliations:** 1 Gene Therapy and Immunogenicity Branch, Division of Cellular and Gene Therapies, Center for Biologics Evaluation and Research, Food and Drug Administration, Bethesda, Maryland, United States of America; 2 Okairòs, Rome, Italy; 3 Centro di Ingegneria Genetica e Biotecnologia Avanzate (CEINGE), Naples, Italy; 4 Department of Biochemistry and Medical Biotechnology, University of Naples Federico II, Naples, Italy; Aaron Diamond AIDS Research Center with the Rockefeller University, United States of America

## Abstract

Among approximately 1000 adenoviruses from chimpanzees and bonobos studied recently, the Pan Adenovirus type 3 (PanAd3, isolated from a bonobo, *Pan paniscus*) has one of the best profiles for a vaccine vector, combining potent transgene immunogenicity with minimal pre-existing immunity in the human population. In this study, we inserted into a replication defective PanAd3 a transgene expressing a fusion protein of conserved influenza antigens nucleoprotein (NP) and matrix 1 (M1). We then studied antibody and T cell responses as well as protection from challenge infection in a mouse model. A single intranasal administration of PanAd3-NPM1 vaccine induced strong antibody and T cell responses, and protected against high dose lethal influenza virus challenge. Thus PanAd3 is a promising candidate vector for vaccines, including universal influenza vaccines.

## Introduction

Influenza continues to pose a global health problem, as highlighted by the 2009 swine influenza pandemic and sporadic human infections with avian H5N1 influenza viruses. Antigenic changes in influenza virus, primarily in the surface antigens hemagglutinin (HA) and neuraminidase (NA), are referred to as antigenic shift (subtype changes by reassortment) and antigenic drift (mutation). This variability among influenza viruses hinders vaccination efforts. Currently, annual surveillance is necessary to identify circulating viral strains for use in vaccine production. New vaccines are often required, and take about 6 months to become available [1]. Thus new approaches are needed.

In contrast, so-called “universal” vaccines targeting relatively conserved components of influenza virus can provide protection regardless of strain or subtype of virus, and may provide an alternative to the use of traditional vaccines. This immunity to conserved antigens would not necessarily prevent infection completely, but might decrease severity of disease, speed up viral clearance, and reduce morbidity and mortality during the initial stages of an outbreak until strain-matched vaccine became available [2]. Furthermore, vaccines based on T cell immunity could be used in combination with a seasonal vaccine to improve efficacy, especially in the elderly who are at high risk of severe disease and show reduced responses to current flu vaccines.

Peptide scanning of T cell responses of healthy human individuals has shown that matrix 1 (M1) and nucleoprotein (NP) are among the prominent targets of CD8^+^ and CD4^+^ T cell cross-recognition [3], so they are of interest as vaccine candidates. By sequence homology, NP is >90% conserved among influenza A isolates [4]. Both murine [5] and human [6] cytotoxic T lymphocytes induced by NP of one virus strain have been shown to cross-react with NP from different influenza A strains. The strong immune responses to NP in mice contribute to protection against challenge [7] via CD8^+^ T cells [5], [8], as well as contributions from CD4^+^ cells [9], [10] and antibodies [11]–[13]. The influenza A matrix (M) gene encodes two highly conserved proteins: an ion channel protein, M2, and the capsid protein, M1. M1 is not a major protective antigen in the mouse and is not well recognized by mouse T cells [14], but has long been known to be recognized by human T cells [15]. Thus its potential contribution to vaccine protection may be underestimated by mouse studies.

While epitopes providing targets widely shared among influenza viruses have been identified in multiple viral proteins, not all of them are highly immunogenic when presented by classical vaccines. More potent immunization can be achieved using recombinant vectors to express the influenza antigens and focus immunity on these targets. Recombinant adenovirus vectors are especially effective at eliciting strong T cell responses to transgene products [16]–[18]. Recombinant adenovirus vectors expressing NP [19] or both NP and M2 [20], [21] can protect mice against a range of influenza virus challenges, including highly pathogenic avian H5N1 strains. While potential interference by prior immunity to human adenoviruses has been suggested as a barrier, this issue can be circumvented by use of vectors based on animal adenoviruses [22]–[25]. Chimpanzee adenoviruses have been shown to be useful vaccine vectors in a variety of animal studies [26]–[30], and the prevalence of neutralizing antibodies against chimpanzee adenoviruses is low in human populations [31]–[33], but not all of them are equally immunogenic.

In this study, we use a simian adenovirus, PanAd3, isolated from the bonobo *Pan paniscus*. This novel adenovirus strain was identified in a study of more than 1000 adenoviruses isolated from chimpanzees and bonobos in order to increase the available repertoire of vectors [34]. In the large scale screening experiments, PanAd3 was among the most potently immunogenic in mice and was also among the least frequently recognized by neutralizing antibodies in human sera.

We have generated a replication incompetent PanAd3 vector deleted of E1 and E3 regions and expressing a fusion protein of the NP and M1 antigens of influenza A, chosen as targets of broad and cross-reactive T cell immunity in humans [3]. The PanAd3-based vaccine was tested for induction of antibody and T cell responses in the systemic and mucosal compartments in mice, as well as for protection against lethal influenza virus challenge. We demonstrate that PanAd3 expressing conserved influenza virus antigens provided highly effective protection after a single intranasal administration. Thus it shows considerable promise as a vaccine candidate.

## Materials and Methods

### Ethics statement

All animal protocols and procedures were approved by the Institutional Animal Care and Use Committee at the Center for Biologics Evaluation and Research (protocol #1991-06) and conducted in an SPF animal facility accredited by the Association for Assessment and Accreditation of Laboratory Animal Care International. All experiments were performed according to institutional guidelines. During influenza challenge studies, animals that had lost 25% of their initial body weight were humanely euthanized to avoid further suffering.

### Influenza viruses

Highly virulent, mouse-adapted virus A/Fort Monmouth/1/47-ma (H1N1) [A/FM] has been previously described [35] and was kindly provided by Earl Brown, University of Ottawa, Canada. It was prepared as a pooled homogenate of lungs from BALB/c mice infected with the virus by the intranasal (i.n.) route 4 days earlier.

### Adenovirus vectors

Pan Adenovirus type 3 (PanAd3) was isolated from a stool specimen collected from a bonobo (*Pan paniscus*). The PanAd3 isolate was amplified and the virus genome was then cloned in a plasmid vector and fully sequenced. As shown in a phylogenetic tree based on hexon sequences [34], PanAd3 is a member of adenovirus species C, closely related to species C human and chimpanzee adenoviruses already used in preclinical and clinical trials (human Ad5, ChAd3).

PanAd3 vectors were constructed by homologous recombination in *E. coli* strain BJ5183 by co-transformation with PanAd3 purified viral DNA and a PanAd3-EGFP shuttle vector. Homologous recombination between pIX genes, right ITR DNA sequences present at the ends of linearized PanAd3-EGFP shuttle and viral genomic DNA allowed its insertion in the plasmid vector, simultaneously replacing the E1 region with a human cytomegalovirus (HCMV) promoter-driven EGFP expression cassette containing the bovine growth hormone polyadenylation signal (BGH polyA), generating pPanAd3ΔE1-EGFP. The E3 region (nucleotides 28684 to 32640) was then deleted through several cloning and homologous recombination steps to generate the pPanAd3ΔE1ΔE3 backbone, which was propagated in HEK 293 cells.

**Figure 1:**
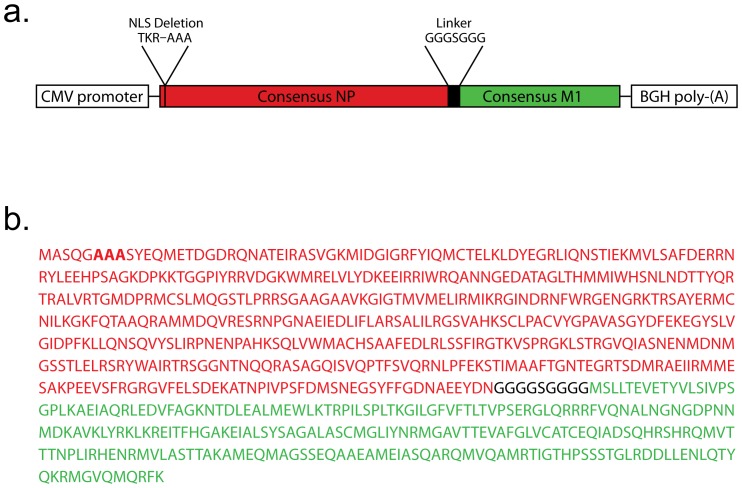
NPM1 fusion protein insert. a) Design of the insert showing CMV promoter, NPM1 transgene, and BGH-polyadenylation cassettes. b) Complete amino acid sequence of the consensus NPM1 fusion protein. NP is indicated in red, linker sequence is shown in black, and M1 is green. The deletion of the nuclear localization signal by mutation of TKR to AAA in NP is indicated in bold text.

Expression cassettes containing consensus sequences of NP and M1 plus the human cytomegalovirus promoter and bovine growth hormone polyadenylation signal were constructed. The influenza expression cassette contains consensus sequences of NP and M1. Influenza A NP and M1 sequences were obtained from the NCBI Influenza Virus Resource database (http://www.ncbi.nlm.nih.gov/genomes/FLU/FLU.html). Protein sequences were chosen from among different subtype strains isolated between 1990 and 2009 that caused infection in humans worldwide. The NP consensus sequence was derived by alignment of all non-identical sequences (H1N1: 88 of 629 sequences, H1N2: 5 of 26, H3N2: 244 of 1557, H5N1: 50 of 121) using MUSCLE version 3.6, and applying the majority rule. Further, the NP sequence used in the PanAd3 vaccine lacks the Nuclear Localization Signal residing in aa 6–8 (TKR mutated to AAA), which results in increased cytoplasmic accumulation. The M1 consensus sequence was similarly derived by the alignment of non-identical sequences (H1N1: 51 of 808 sequences, H1N2: 3 of 34, H3N2: 115 of 2150, H5N1: 23 of 145). NP and M1 sequences were spaced by a flexible linker (GGGSGGG). The resulting NPM1 sequence was codon-optimized for expression in eukaryotic cells. A diagram of the insert and its full sequence are given in Figure 1. The NPM1 expression cassette was inserted into the PanAd3ΔE1ΔE3 backbone via homologous recombination in *E.coli*. Sequences for HIV gag protein or a respiratory syncytial virus (RSV) fusion protein of F protein, nucleoprotein N and transcription factor M2-1 were inserted in constructs to be used as specificity controls. Expression cassettes were inserted into a pNEB shuttle vector and then transferred into the SnaBI linearized pPanAd3ΔE1ΔE3-EGFP plasmid by homologous recombination in *E. coli*, exploiting the homology between the HCMV promoter and BGH polyA sequences. The PanAd3 vectors were produced in Procell 92 cells, which were derived from the HEK 293 cell line originally banked at the University of Leiden in 1973 [36] and obtained from Frank Graham at MacMaster University (Hamilton, Canada), and further adapted at Okairòs to be suitable for manufacturing by incorporation of a plasmid carrying a Tet repressor expression cassette and G418-resistance gene. The protocol for generating the Procell 92 cell line followed essentially that published by Matthews et al. [37]. Briefly, HEK 293 cells were transfected with an expression vector containing a cassette encoding the Tet repressor under control of the human phosphoglycerate kinase-1 (PGK) promoter, and the G418-resistance gene. Single clones were selected by growing the transfected cells in the presence of 1 mg/ml G418 in culture medium. Single clones were amplified and tested for Tet repressor expression by Western Blot analysis. The stability of Tet repressor expression in the selected clone was tested up to passage 63. PanAd3 vectors grown in these cells were purified by cesium chloride gradients and stored in buffer A195 [38].

Viral particle (vp) measurements of adenovirus stocks were made by measurement of absorbance at 260 nm as described [39].

### Peptides and proteins

Peptides NP_147–155_ (TYQRTRALV) and SARS M_209–221_ (HAGSNDNIALLVQ) were synthesized by the CBER core facility. An MHC-I restricted peptide of adenovirus DNA-binding protein (Dbp_419–427_: FALSNAEDL), present in PanAd3 [40] and recombinant M1 (rM1) protein from strain A/PR/8/34 (H1N1) were purchased from Genscript (Piscataway, NJ). Recombinant nucleoprotein (rNP) from strain A/PR/8/34 (H1N1) was purchased from Imgenex (San Diego, CA).

### In vitro expression and Western blot (WB)

HeLa cells were infected with PanAd3-NPM1 at indicated multiplicities of infection (MOI). Extracts were prepared 48 hours after infection using TEN buffer (20 mM Tris pH 7.5, 150 mM NaCl, 1 mM EDTA pH 8, 1% Triton X100 and protease inhibitors). Nuclei and cell debris were spun out by centrifugation at 7,500 g, 30 minutes at 4°C. Glycerol was added to supernatants to 10% and stored at −20°C. Expression was assessed by Western blotting with a mouse hyperimmune serum raised against the NPM1 antigen.

### Mouse immunization and challenge infection

Female BALB/cAnNCr mice aged 5–7 weeks were purchased from the National Cancer Institute, Frederick, MD. Inoculations were performed by administration of 50 µl vaccine or challenge virus given i.n. while mice were under isoflurane anesthesia, or by administration of 100 µl of vaccine by intramuscular (i.m.) injection. Mice were immunized at 10 weeks of age with indicated doses. Some groups of mice were challenged 4 weeks post-immunization under isoflurane anesthesia with 10^4^ TCID_50_ (100 LD_50_) of A/FM.

### Mucosal sampling

Mice were euthanized and bronchoalveolar lavage (BAL) fluid and lung cells obtained as in Price et al., 2009 [20]. Briefly, for BAL fluid, lungs were flushed with 1 ml phosphate-buffered saline (PBS). Lung cells were isolated by gradient centrifugation of minced and collagenase-digested lung tissue.

### Spleen and blood sampling

Splenocytes were depleted of erythrocytes by treatment with ACK lysis buffer. Sera from blood collected from the abdominal vena cava were isolated using BD Microtainers (Franklin Lakes,NJ), and decomplemented by heat-treating at 56°C for 30 minutes.

### T cell ELISPOT

T cell ELISPOT assays were performed as described previously [41]. Briefly, anti–interferon (IFN)-γ mAb AN18 (BD Pharmingen, San Jose, CA) was used to coat ELISPOT plates (Millipore, Billerica, MA). Splenocytes or lung cells were added to wells at a concentration of 250,000 cells/well (and, when necessary, also 62,500 cells/well to bring results into the countable range) in CT medium [42]. Peptides were added at a final concentration of 1 µg/ml. Plates were incubated for 36–48 hr at 37°C in 5% CO_2_. Bound IFN-γ was detected with biotinylated mAb R4–6A2 (BD Pharmingen) followed by incubation with alkaline phosphatase–labeled streptavidin (KPL, Gaithersburg, MD). 100 µl 5-bromo, 4-chloro, 3-indolylphosphate/nitroblue tetrazolium was used as the developing substrate (KPL). Spots were counted with an ELISPOT reader (Zeiss; Thornwood, NY).

### Antibody analysis

Antibody levels in serum and BAL were measured by enzyme-linked immunosorbent assay (ELISA) as in Benton et al. [43]. Specifically, NUNC 96-well plates were coated at 4°C overnight with 1 µg/ml of rNP or 5 µg/ml of rM1 in PBS, then blocked. Next individual mouse sera or BAL were added to the plates. Bound antibody was detected using human-adsorbed alkaline phosphatase-conjugated goat anti-mouse IgG, or IgA (Southern Biotechnology Associates, Birmingham, AL) followed by the substrate *p*-nitrophenyl phosphate (Sigma). OD was measured at 405 nm.

### Neutralizing antibody assay

Ad5 and PanAd3 neutralizing antibody titers were assayed as previously described [31] with some modifications. Briefly, 3.5×10^4^ HEK293 cells per well were seeded in a 96 well plate and cultured for 2 days. Each adenoviral vector expressing secreted alkaline phosphatase (SeAP) was incubated for 1 hour at 37°C alone or with serial dilutions of serum, and then added to the 95–100% confluent HEK293 cells and incubated for 1 hour at 37°C. Supernatant was then removed and replaced with 10% FCS in DMEM. SeAP expression was measured 24 hours later using the chemiluminescent substrate (CSPD), from the Phospha-LightTM kit (Tropix Cat No T1016, Applied Biosystems, Bedford, MA) without heat inactivation. Light emission (relative light units, RLU) was monitored 45 minutes after the addition of the CSPD substrate, using the Envision 2102 Multi-label reader (Perkin Elmer, Waltham, MA).

### Statistical analysis

Survival data for vaccine groups vs. controls were compared by Log-Rank analysis and the Bonferroni Method using PRISM (GraphPad Software, Inc., La Jolla, CA).

## Results

### Expression of influenza proteins from PanAd3 vectors

**Figure 2:**
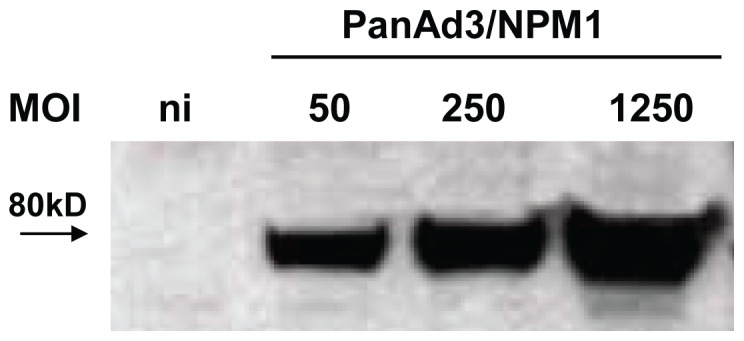
Detection of influenza antigens expressed by vectors in cultured cells. Western blot analysis of HeLa extracts prepared as described after 48 hours infection with PanAd3NPM1 at 50, 250, 1250 MOI (ni = not infected). The blotted proteins were revealed with a mouse hyperimmune serum raised against the NPM1 antigen. The 80 kD major band is consistent with the fusion NPM1 protein.

The PanAd3-NPM1 construct was designed using two conserved influenza antigens important in human immunity, NP and M1. To analyze the level of transgene expression, HeLa cells were infected with PanAd3-NPM1 at various MOI, and Triton extracts prepared. Western blot analysis of the extracts was performed using a mouse hyperimmune serum raised against the NPM1 antigen. The 80 kD major band seen is consistent with the fusion NPM1 protein (**Fig. 2**). The 80 kD band was also detected if the Western blot was developed with a monoclonal antibody to NP (data not shown).

### Immune responses to PanAd3-NPM1, comparing i.m. and i.n. routes

Intranasal (i.n.) immunization is especially efficient for induction of local immunity in the respiratory tract, including recruitment of memory T cells to the airways [44]. For a given vaccination, i.n. induces greater mucosal immune responses than intramuscular (i.m.) immunization, but weaker systemic responses [20], [21]. In pilot studies, we included both routes of immunization, in order to characterize the responses induced by the new vector.

#### Antibody responses

**Figure 3:**
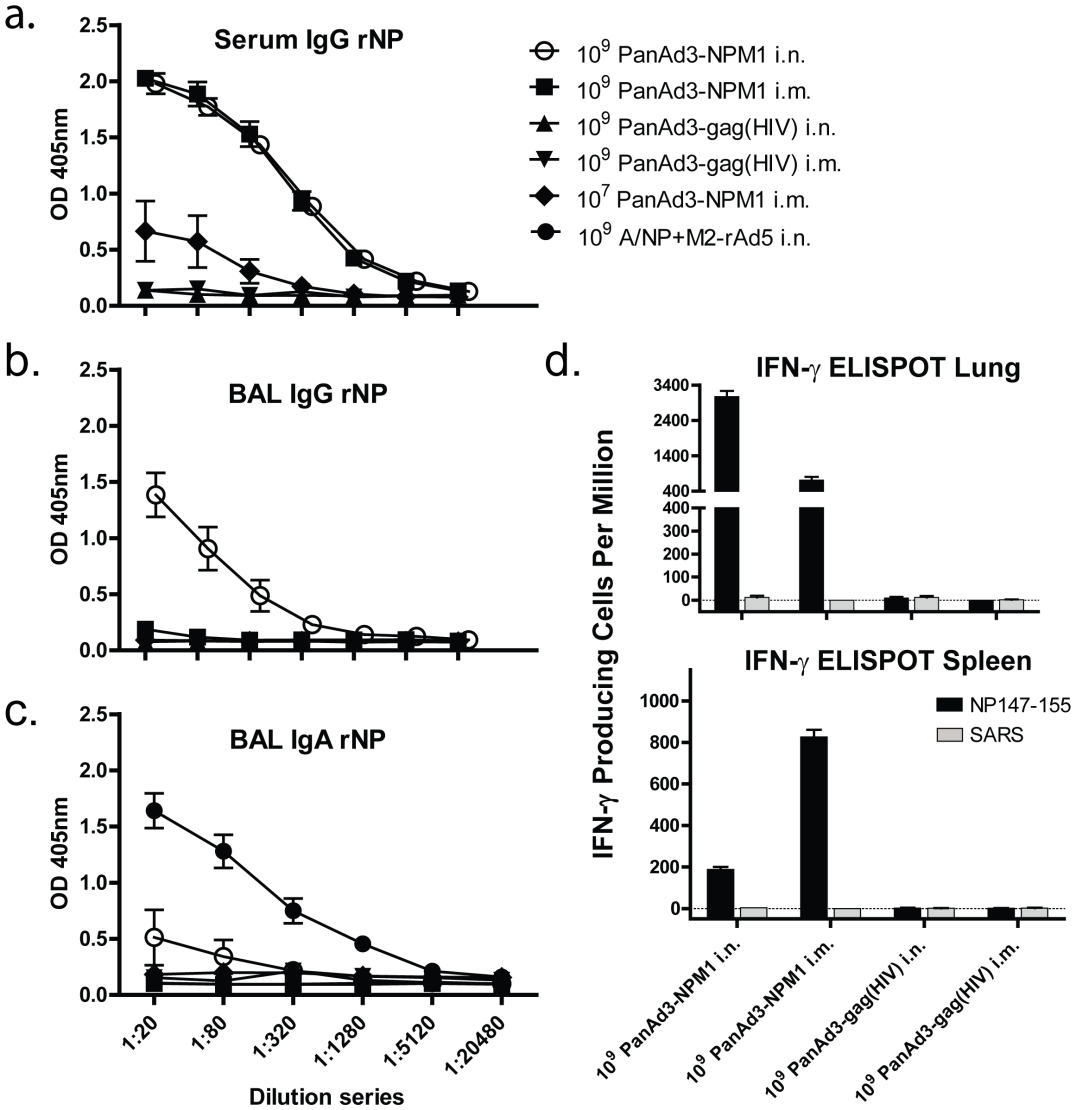
Antibody and T cell responses to PanAd3NPM1. Mice were vaccinated with 10^9^ or 10^7^ total vp/mouse. Immunizations were by either the i.n. or i.m. route, as indicated. Four weeks later mice were sacrificed, and serum and BAL were collected for antibody analysis. Lung T cells were also collected for IFN-γ ELISPOT analysis. Error bars indicate mean ± SEM. a) ELISA for IgG antibodies to rNP in the serum. b) ELISA for IgG antibodies to rNP in the BAL. c) ELISA for IgA antibodies to rNP in the BAL. d) ELISPOT for IFN-γ T cell responses. Results for three mice per group are reported as number of IFN-γ secreting cells per 10^6^ cells. Black bars, stimulation with NP_147–155_ peptide. Gray bars, stimulation with SARS_209–221_ peptide. Top: T cells in the lungs. Bottom: T cells in the spleen. Bars show mean ± SEM of pooled group triplicates, not individual animals.

Sera from individual mice 4 weeks after immunization were tested for antibodies to NP. As shown in **Figure 3A**, equivalent IgG responses to NP were detected in serum responses to PanAd3-NPM1 given either i.m. or i.n. at a dose of 10^9^ viral particles (vp). Serum antibody responses were reduced when animals were immunized with a lower dose (10^7^ vp) of PanAd3-NPM1. In the BAL, anti-NP IgG antibodies were induced by i.n. but not i.m. immunization (**Fig. 3B**). PanAd3-NPM1 induced very little IgA in the BAL (**Fig. 3C**). A reagent control provided by BAL from A/NP+M2-rAd5 immunized mice, a system known from previous studies to induce IgA [21], showed that the assay could detect IgA antibodies if present.

#### T cell responses

Functional T cell responses to vaccination were measured by IFN-γ ELISPOT. **Figure 3D** shows responses in the spleen and lungs to NP_147–155_ peptide, the immunodominant MHC I epitope of CD8^+^ T cells in BALB/c mice [45]. Immunization with PanAd3-NPM1 i.m. produced much higher frequencies of NP-specific T cells in the spleen than i.n. immunization, while the reverse was true in the lungs. These results show anatomical localization of the immune response, with i.n. more efficiently priming T cells in the respiratory tract, consistent with previous studies [20], [21], [44]. No response to NP was seen in mice immunized with constructs containing an irrelevant transgene (HIV gag), and none of the mice responded to the SARS_209–221_ control peptide.

A pilot experiment showed protection against challenge four weeks post-vaccination with 10^9^ vp of PanAd3-NPM1 given i.n. (data not shown). Thus the PanAd3 vector was promising, and we pursued more detailed studies.

### Detailed characterization of immune responses to mucosally administered PanAd3 recombinant

Given the superiority of i.n. administration for inducing T cell responses in the lungs, we further explored the immune responses to vaccination by this mucosal route, using PanAd3-NPM1 or as a control PanAd3 with an irrelevant RSV insert. Mice were immunized with doses of 10^9^,10^7^, or 10^5^ vp per mouse.

#### Antibody responses

**Figure 4:**
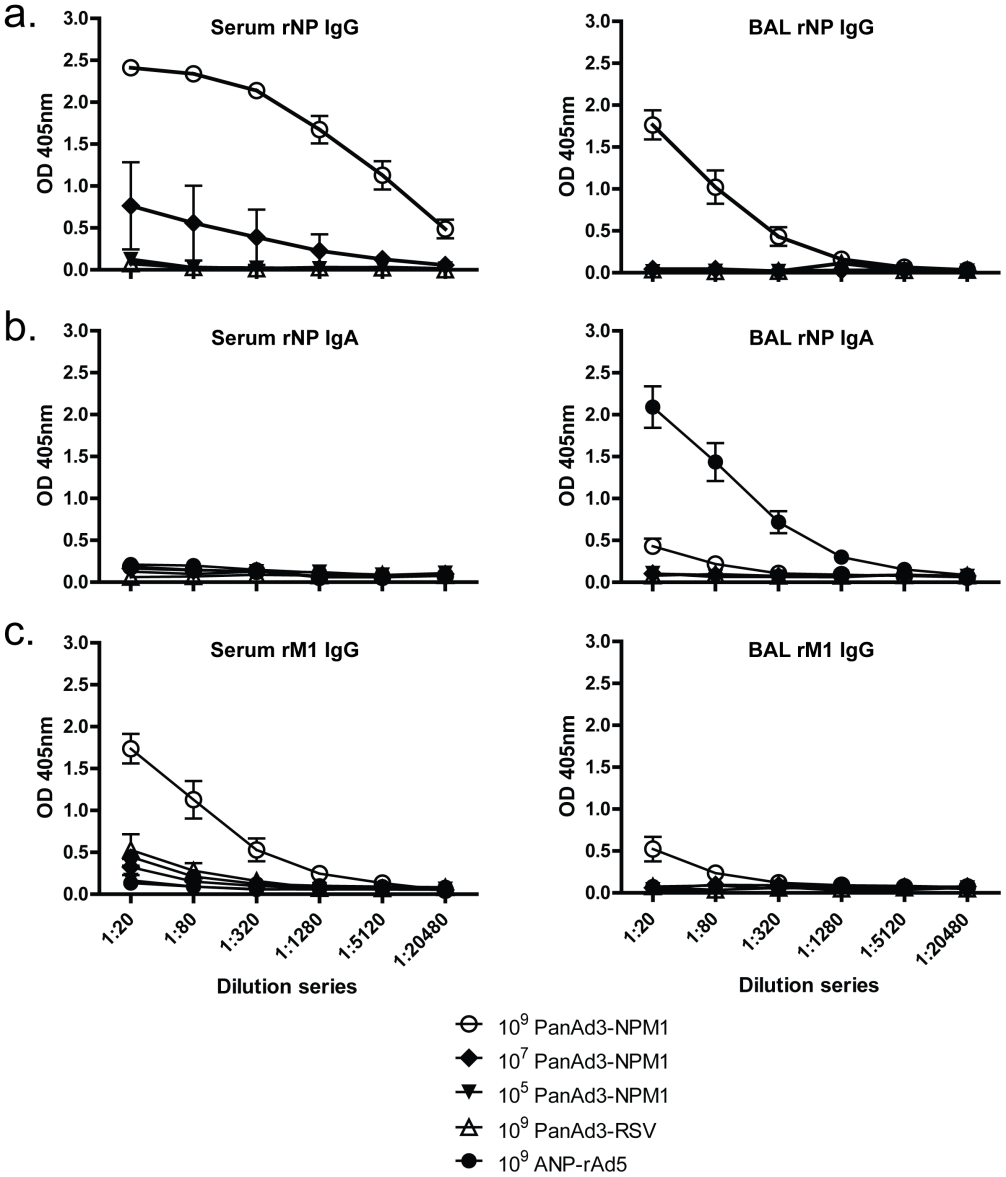
Antibody responses to different doses of PanAd3NPM1. Mice were vaccinated with indicated doses. Four weeks later mice were sacrificed, and serum and BAL were collected for antibody analysis. Error bars indicate mean ± SEM. a) ELISA for IgG antibodies to rNP in the serum and BAL. b) ELISA for IgA antibodies to rNP in the serum and BAL. c) ELISA for IgG antibodies to rM1 in the serum and BAL.

Serum and BAL were analyzed for IgG and IgA antibodies to NP and M1. **Figure 4A** shows results for IgG antibodies to NP in serum and BAL. At the highest vaccine dose, 10^9^ vp per mouse, strong IgG responses were seen for PanAd3-NPM1. If the vaccine dose given to the mice was reduced to 10^7^ vp per mouse, antibody responses were greatly reduced in serum and absent in BAL.

The IgA antibody response to PanAd3-NPM1 was undetectable in serum and marginal in BAL (**Fig. 4B**). As in Figure 3, a reagent control provided by BAL from A/NP-rAd5 immunized mice showed that the assay could detect IgA antibodies if present. The antibody response to the M1 component of PanAd3-NPM1 did not include IgA (data not shown) and the IgG response to M1 was only substantial in the serum (**Fig. 4C**).

#### T cell responses

**Figure 5:**
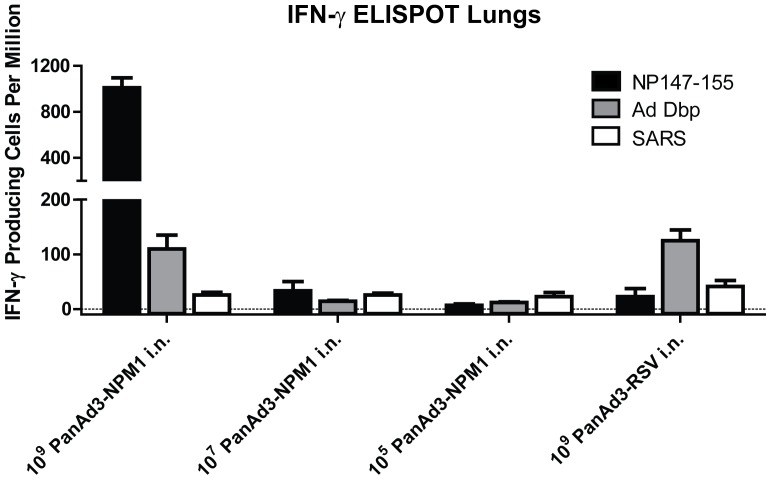
T cell responses to different doses of PanAd3-NPM1. Mice were immunized as in **Figure 3**. Four weeks post-immunization, T -cells of three mice per group were assayed by IFN-γ ELISPOT. Results shown are for lung T cells, and are reported as number of IFN-γ secreting cells per 10^6^ cells. Bars show mean ± SEM of results for lungs of individual mice.

T cell responses were again measured by IFN-γ ELISPOT. At a dose of 10^9^ vp, PanAd3-NPM1 induced a strong T cell response in the lungs to the dominant NP_147–155_ epitope. Both PanAd3-NPM1 and the PanAd3-RSV control induced modest responses to the adenovirus peptide Dbp_419–427_ (**Fig. 5**). A hundred-fold lower dose of PanAd3-NPM1 induced little response to either NP_147–155_ or Dbp_419–427_.

#### Immune protection against challenge infection

**Figure 6:**
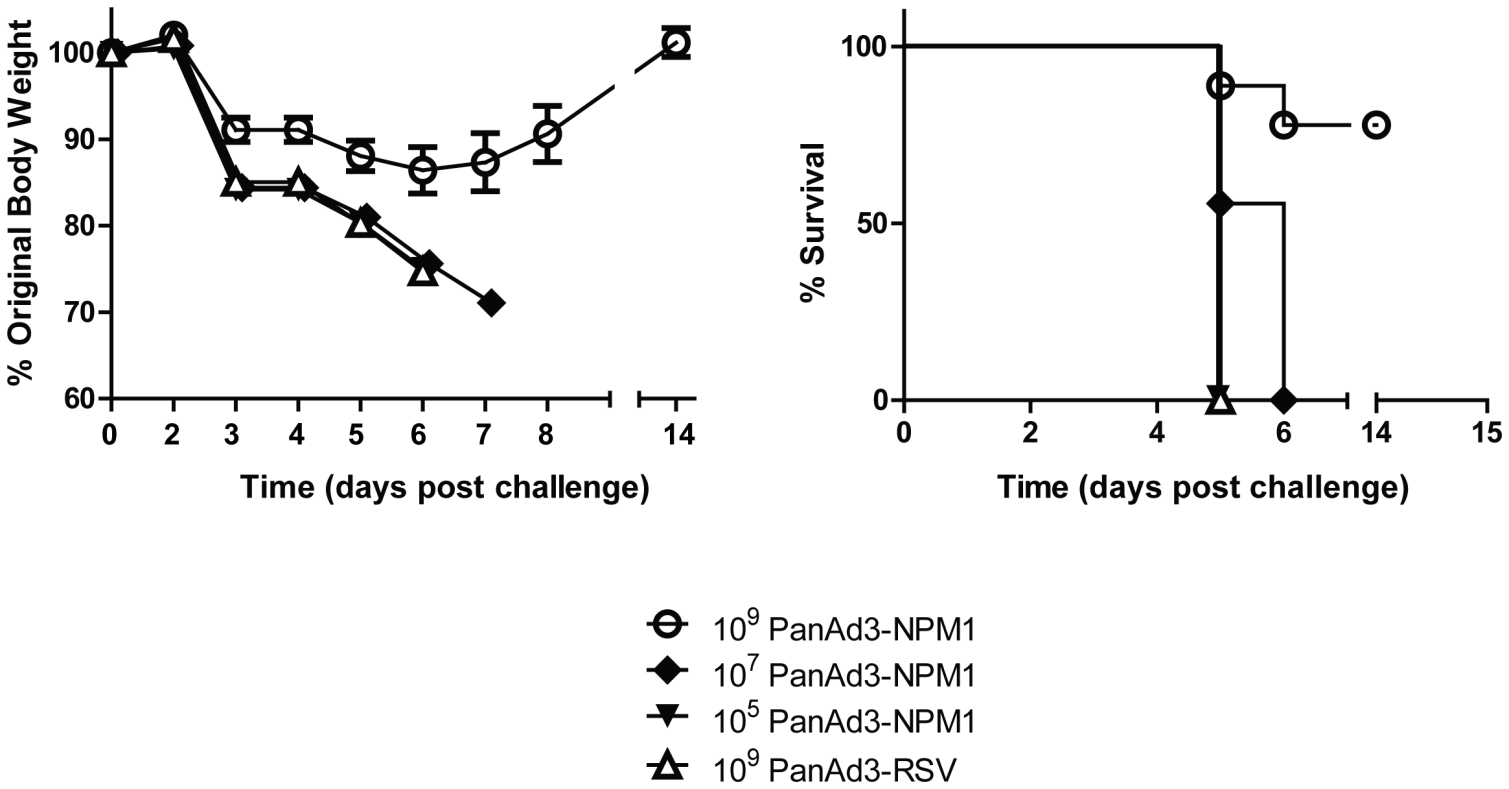
Protection against lethal influenza challenge by PanAd3-NPM1. Mice were immunized as in **Figure 3**. Approximately 9 weeks post-immunization, mice were challenged with influenza virus A/FM at a dose of 10^4^ TCID_50_ (100 LD_50_) per mouse, and monitored for body weight and mortality. Survival of the PanAd3-NPM1 group at the dose of 10^9^ vp differs significantly (p<0.05) from the PanAd3-RSV control group. Error bars indicate mean ± SEM.

One month after immunization mice were challenged with 100 LD_50_ (10^4^ TCID_50_) of A/FM, a relatively high dose of a highly pathogenic virus. As shown in **Figure 6**, a dose of 10^7^ vp or less did not protect, but at a dose of 10^9^ vp PanAd3-NPM1 provided considerable protection against this stringent challenge. Most mice survived and displayed much less morbidity than controls, as shown by weight loss.

#### Cross-neutralization by human antibodies to Ad5

Ad5 and PanAd3 are closely related viruses, both belonging to adenovirus group C [34]. As one aspect of whether PanAd3 vectors are likely to be blocked by pre-existing immunity to Ad5, we tested neutralization of a PanAd3 virus by human sera selected for particularly high neutralizing antibody to Ad5 (titers >1000). As shown in Table S1, many of these high-titered sera had no neutralizing activity on PanAd3. Some of the human sera with very high neutralizing titers ranging from 1628 to 4608 on Ad5 had low neutralizing titers of 28–63 on PanAd3.

## Discussion

In the efforts to develop a universal influenza vaccine, various platforms for immunization to conserved antigens have been studied. Replication incompetent adenovirus vectors are promising, since their strong induction of innate immune responses provides a built-in adjuvant, and the antigen-specific B and T cell responses they induce are sustained for a long time [21]. Animal adenoviruses have the potential advantage that humans have no prior exposure to them. For that reason chimpanzee adenoviruses have recently begun to be explored for use as vaccine vectors in humans, where they showed good safety and excellent immunogenicity [27], [29], [46]. Furthermore, in tests of Ad5 and four chimpanzee adenovirus vectors, prior immunization with a GFP-expressing construct blocked subsequent responses to the transgene product only for homologous vector; cross-blocking was minimal [34].

In this study, we tested a new vector based on bonobo virus PanAd3. Both Ad5 and bonobo virus PanAd3 belong to the adenovirus species C [34]. Species B (for example Ad35) has been shown in other studies to be less immunogenic than species C [47]. Since they are so closely related, Ad5 and PanAd3 may share certain structural features providing powerful internal adjuvant effects and thus higher immunogenicity. Despite the structural similarity, human sera containing high anti-Ad5 neutralization titers (>1000) show no or marginal neutralization capacity on PanAd3. Moreover, in a screening study, PanAd3 was shown to be a potent vector in mice and in primates [34]. We show here that a single administration of PanAd3-NPM1 influenza vaccine given i.n. provided highly effective protection against lethal challenge with mouse-adapted A/FM, with greatly reduced morbidity and mortality compared to controls. Protection was comparable to that in previously published studies of Ad5 expressing conserved influenza virus antigens for the same challenge virus and dose [20], [21].

The PanAd3 vaccine induced both antibody and T cell responses to NP. As mentioned earlier, the T cell response to NP is well-known to contribute to protection [5], [8]–[10], and recent studies have reported that antibodies to NP can also contribute to protection [12].

The fusion protein of NP with M1 expressed by the PanAd3 vaccine has the advantage of including another major target of human immunity. Using multiple target antigens may invoke different immune mechanisms, reduce the likelihood of generating escape mutants, and provide a larger range of epitopes that may be suitable for different MHC types. Although M1 is not expected to play much of a role in protection in mice, it is a prominent target of T cell immunity in humans [3], and might contribute to the performance of the PanAd3-NPM1 vaccine in humans.

The results presented here support the use of the PanAd3 vector as a vaccine candidate that is highly effective at inducing T cell and antibody immunity, while at the same time having the advantage that it is not neutralized by human sera [34]. Thus PanAd3, when used to express conserved influenza virus antigens, has promise as a “universal” influenza vaccine candidate.

## Supporting Information

Table S1 **Sera from healthy human individuals from different geographical areas in Europe and the United States had been screened previously for neutralizing activity to Ad5** [**34**] **.** Selected sera with high Ad5 neutralizing activity (titers >1000) were tested for neutralization of PanAd3 as described in Materials and Methods, using vectors expressing the secreted alkaline phosphatase (SeAP) reporter gene. * Arbitrary sample numbers. ** Results of two tests. Ethics statement: All volunteers gave written informed consent before participation, and the studies were conducted according to the principles of the Declaration of Helsinki and in accordance with Good Clinical Practice.
(DOC)
