# Supplementary material for: Vaccination to Conserved Influenza Antigens in Mice Using a Novel Simian Adenovirus Vector, PanAd3, Derived from the Bonobo Pan paniscus
Source: PLoS One. 2013 Mar 11;8(3):e55435. doi: 10.1371/journal.pone.0055435 (PMC3594242; doi:10.1371/journal.pone.0055435)
Supplement: Table S1 — Sera from healthy human individuals from different geographical areas in Europe and the United States had been screened previously for neutralizing activity to Ad5 [34] . Selected sera with high Ad5 neutralizing activity (titers >1000) were tested for neutralization of PanAd3 as described in Materials and Methods, using vectors expressing the secreted alkaline phosphatase (SeAP) reporter gene. * Arbitrary sample numbers. ** Results of two tests. Ethics statement: All volunteers gave written informed consent before participation, and the studies were conducted according to the principles of the Declaration of Helsinki and in accordance with Good Clinical Practice. (DOC) [file pone.0055435.s001.doc]

Supplementary Table 1.

| Serum* | Neutralizing titer on Ad5 | Neutralizing titer on PanAd3 |
| --- | --- | --- |
| 1 | 1271 | <18 |
| 2 | 2186 | <18 |
| 3 | 1712 | <18 |
| 4 | 3408 | <18 |
| 5 | 3206 | <18 |
| 6 | 2962/2473** | <18 |
| 7 | 2528 | <18 |
| 8 | 294/230** | <18 |
| 9 | 2280 | <18 |
| 10 | 2372 | <18 |
| 11 | 3425 | <18 |
| 12 | >4608 | <18 |
| 13 | >4608 | <18 |
| 14 | >4608/>4608** | 42 |
| 15 | >4608 | 42 |
| 16 | >4608 | 63 |
| 17 | 2027 | <18 |
| 18 | 3297 | <18 |
| 19 | 1141 | <18 |
| 20 | 2051 | 29 |
| 21 | 2357 | 52 |
| 22 | 3893 | <18 |
| 23 | 1628 | 28 |
| 24 | 2447 | 34 |
| 25 | 2407 | 40 |
| 26 | 2736 | <18 |
| 27 | 3848 | 39 |
